# Discovery of genomic variation across a generation

**DOI:** 10.1093/hmg/ddab209

**Published:** 2021-07-22

**Authors:** Brett Trost, Livia O Loureiro, Stephen W Scherer

**Affiliations:** The Centre for Applied Genomics and Program in Genetics and Genome Biology, The Hospital for Sick Children, Toronto, ON M5G 0A4, Canada; The Centre for Applied Genomics and Program in Genetics and Genome Biology, The Hospital for Sick Children, Toronto, ON M5G 0A4, Canada; The Centre for Applied Genomics and Program in Genetics and Genome Biology, The Hospital for Sick Children, Toronto, ON M5G 0A4, Canada; McLaughlin Centre and Department of Molecular Genetics, University of Toronto, Toronto, ON M5S 1A8, Canada

## Abstract

Over the past 30 years (the timespan of a generation), advances in genomics technologies have revealed tremendous and unexpected variation in the human genome and have provided increasingly accurate answers to long-standing questions of how much genetic variation exists in human populations and to what degree the DNA complement changes between parents and offspring. Tracking the characteristics of these inherited and spontaneous (or *de novo*) variations has been the basis of the study of human genetic disease. From genome-wide microarray and next-generation sequencing scans, we now know that each human genome contains over 3 million single nucleotide variants when compared with the ~ 3 billion base pairs in the human reference genome, along with roughly an order of magnitude more DNA—approximately 30 megabase pairs (Mb)—being ‘structurally variable’, mostly in the form of indels and copy number changes. Additional large-scale variations include balanced inversions (average of 18 Mb) and complex, difficult-to-resolve alterations. Collectively, ~1% of an individual’s genome will differ from the human reference sequence. When comparing across a generation, fewer than 100 new genetic variants are typically detected in the euchromatic portion of a child’s genome. Driven by increasingly higher-resolution and higher-throughput sequencing technologies, newer and more accurate databases of genetic variation (for instance, more comprehensive structural variation data and phasing of combinations of variants along chromosomes) of worldwide populations will emerge to underpin the next era of discovery in human molecular genetics.

## Introduction

**Figure 1:**
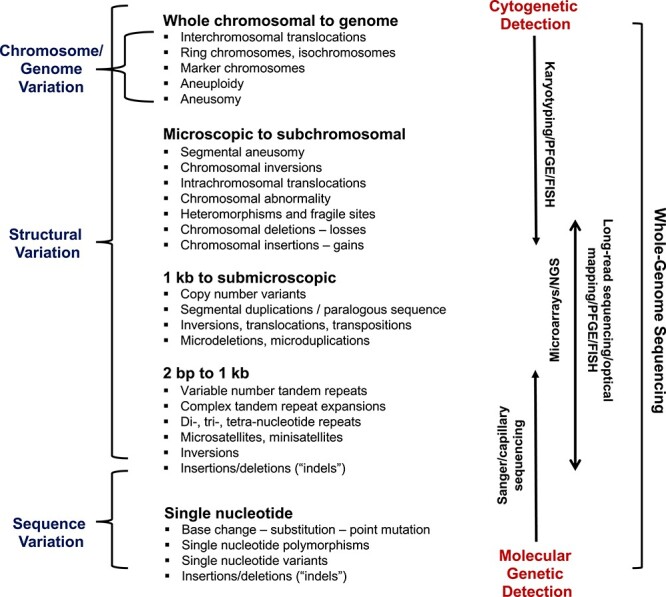
Types of variation found in the human genome and the primary technologies used to detect them (43). The types of variation, and various (sometimes synonymous) terms used to describe them, are grouped as ‘sequence variation’ and ‘structural variation’, the latter encompassing chromosomal/genome variation. The lower end-size of structural variation is typically defined to fall in the 50–1000 nt range, but definitions vary (9,172). FISH, fluorescence *in situ* hybridization (here also encompassing spectral karyotyping); PFGE, pulse field gel electrophoresis; NGS, next-generation sequencing (including both short-read and long-read technologies, the latter being particularly useful for identifying intermediate-size structural variation). There are many other important technologies used to discover and map genetic variation and we include those that have been most impactful for the original discoveries discussed in this review, including those that are still used by clinical diagnostic laboratories. Important references are provided in Tables 1 and 2 and the main text.

Perhaps the greatest paradigm shift for genetics research in recent years has been the move from analyzing just one gene at a time to being able to interrogate the entire genome at once—every gene, be it coding or non-coding, along with all the DNA in between (1–3). Driven by extraordinary innovations in laboratory technology and information sciences, this advance has led to the (re)-birth of the field of genomics (4), particularly as it impacts health care (5). We consider it a re-birth because, from the earliest studies of chromosomes 60–70 years ago, the first direct vantage point of genetics was the morphological anatomy of the genome, not the gene (6–8). As summarized in Figure 1, the classes of genetic variation being described at that time (e.g. aneuploidies; large translocations and deletions) were those that could be seen from cytogenetically stained chromosomes. Although higher-resolution banding eventually enabled the detection of subtler changes, all of these experiments were inextricably linked to the limits of microscopic observation (9).

Modern genomics arguably began with the elucidation of the structure of DNA in the 1950s (10) and the determination of the genetic code and the modern concept of the gene in the 1960s (11,12). The next three decades saw the development of a plethora of revolutionary DNA sequencing and recombinant DNA cloning technologies that allowed the decoding of individual genes at the nucleotide level, leading to the identification of point mutations and more complex di-, tri- and tetra-nucleotide variants (13–15). Together, the new genomics technologies consolidated genetic (14,16,17) and physical linkage (18,19) strategies and provided the basis for generating the first holistic descriptions of chromosomes and the genome. The decade bridging the year 2000 brought forward chromosomal microarray analysis [CMA; (20–26)], which afforded truly global genotyping capability, including assessment of submicroscopic deletions and duplications in disease samples, as well as the discovery of a previously unrealized amount of DNA copy number variation (CNV) in all individuals (27–29). Moreover, the implementation of automated fluorescence-based DNA sequencing, including clone-end and full-clone ‘shotgun’ sequencing, led to the 2001 release of working draft assemblies of the human genome (1,2), with the first ‘full’ reference sequence, denoted GRCh35, published in 2004 (3). The availability of a high-quality reference assembly provided an entry point for concurrent personal genome sequencing and the generation of integrated maps of genetic variation (30). Recognition of the importance of accurate human genome sequencing at scale led to the (ultimately canceled) $10M ‘Archon Genomics X PRIZE’ to the first group able to sequence haplotype-resolved genomes satisfying what turned out to be then (and still remain) unreachable criteria for cost and accuracy (31). Perhaps the single most important technology underpinning the current state of genomics is massively parallel DNA sequencing, which was first developed in the late 2000s (32–36). These ‘next-generation sequencing’ (NGS) technologies can be used to study the human genome at population scale with unprecedented resolution. Augmented by NGS, the latest release of the human reference genome, GRCh38, includes over 97 million more sequenced bases than GRCh35 (3,37–39).

In formulating this review, we aimed to examine two questions fundamental to our understanding of human genetics and its application to medicine—namely, how much variation exists in our diploid genome, and with this baseline, how does its nucleotide composition change from one generation to the next? At the inauguration of the important journal *Human Molecular Genetics* some 30 years ago, having (mostly) accurate answers to these vital questions would have seemed unattainable. *Circa* 2021, however, for the historically well-studied chromosomal- and sequence-level variation, this information is nearing perfection, at least in most euchromatic DNA. In contrast, data for intermediate-sized structural variation (9,40–46), the last broad class of variation to be characterized (Fig. 1), are now catching up as new technologies and algorithms are developed (47,48).

## Genetic Variation at the Level of the Individual Human

In 2001, two separate groups, the International Human Genome Sequencing Consortium and Celera Genomics, published initial haploid drafts of the human genome. Both sequences were derived from composites of individuals, and they were generated using highly automated fluorescence-based Sanger DNA sequencing (49) from clone-based and random whole-genome sequencing (WGS), respectively (1,2). In 2007, the ‘HuRef’ genome—the first genome sequence of an individual human (Craig Venter)—was assembled (50), providing a pivotal starting point to query how much genetic variation exists within a ‘diploid’ human genome. For this once-in-a-generation project, which built upon Celera Genomics’ original efforts (and cost ~$70M), ~ 1000 bp reads were generated from over 30 million random DNA fragments using Sanger sequencing. These reads were then assembled into 4528 scaffolds, with the assembly strategy enabling alternate alleles in the diploid genome to be defined.

Comparison of this accurate assembly to the reference genome of the time revealed 3 213 401 single nucleotide variants (SNVs) and 851 575 insertions/deletions (indels), which collectively encompassed 12.3 Mb of DNA (Table 1). The observation that non-SNV variants comprised 22% of events in HuRef but 74% of modified base pairs, implying a substantial contribution of larger genetic variants to overall variation, set the standard for how future personal genomes might be characterized, irrespective of the technology used. Further analysis of the HuRef assembly, combined with CMA (22,23), identified 12 178 structural variants (SVs); combined with the non-SNV alterations identified in the initial study, this yielded an estimated total of 39.5 Mb of non-SNV unbalanced variation, along with 90 inversions encompassing 9.3 Mb (51). Thus, the HuRef genome differs from the reference by only ~ 0.1% when considering SNVs alone, but by a far larger amount (~1.3%) when considering all forms of unbalanced variation. A compelling lesson from this and other early studies of the human genome was that no single sequencing (or other) technology could accurately reveal all of the classes of genetic variation shown in Figure 1 (52).

**Table 1:** Important WGS studies examining the extent of variation in a genome^a^

						Indels		CNVs/SVs			Non-SNV variation (Mb)^b^		
Study	Genome	Ancestry^c^	Sex	DNA	SNVs	Ins	Del	Ins/Dup^d^	Del	Inv	Unbalanced	Balanced	Technologies^e^
Levy *et al.* 2007 *PloS Biol.* (50)	HuRef (Venter)	EUR	M	Blood	3 213 401	275 512^f^	283 961^f^	30	32	90	–	–	CMA, SS
Pang *et al.* 2010 *Genome Biol.* (51)					–	412 304^g^	383 775^g^	9915^g^	13 867^g^	167	39.5	9.3	
Wheeler *et al.* 2008 *Nature* (53)	Watson	EUR	M	Blood	3 322 093	65 677	157 041	9	14	–	–	–	454, CMA
Lupski *et al.* 2010 *N. Eng. J. Med.* (54)	III-4 (Lupski)	EUR	M	Blood	3 420 306	–	–	123	111	–	–	–	CMA, SOLiD
Ebert *et al.* 2021 *Science* (124)	NA12878	EUR	F	LCL	3 643 864	367 945	372 590	13 954	8931	108	15.3	21.7	PB, S-seq
Bentley *et al.* 2008 *Nature* (34)	NA18507	AFR	M	Blood	4 139 196	176 221	228 195	2345	5704	–	–	–	IL
Schuster *et al.* 2010 *Nature* (55)	KB1	AFR	M	Blood	4 053 781	–	–	–	–	–	–	–	454, IL
Ebert *et al.* 2021 *Science* (124)	HG03125	AFR	F	LCL	4 470 531	438 853	449 021	16 355	10 775	120	17.5	22.2	PB, S-seq
Chaisson *et al.* 2019 *Nat. Commun.* (141)	NA19240	AFR	F	LCL	–	419 842^h^	370 245^h^	17 026	12 421	129^i^	39.8	19.6^i^	10X, BN, Hi-C, IL, ON, PB, S-seq
Kim *et al.* 2009 *Nature* (56)	AK1	EAS	M	Blood	3 453 653	75 141	95 061	581	656	–	–	–	CMA, IL
Seo *et al.* 2016 *Nature* (58)				LCL	3 472 576	169 314 total		10 077	7358	71	13.6^j^	13.5	10x, BN, IL, PB
Shi *et al.* 2016 *Nat. Commun.* (63)	HX1	EAS	M	Blood	3 518 309	16625 690 total		10 284	9891	–	11.0^j^	–	BN, IL, PB
Ebert *et al.* 2021 *Science* (124)	HG00512	EAS	M	LCL	3 620 202	367 796	370 030	14 055	8937	122	15.5	21.0	PB, S-seq
Chaisson *et al.* 2019 *Nat. Commun.* (141)	HG00514	EAS	F	LCL	–	335 762^h^	297 565^h^	15 566	10 291	121^i^	39.3	14.1^i^	10X, BN, Hi-C, IL, ON, PB, S-seq
Takayama *et al.* 2021 *Nat. Commun.* (64)	JG1^k^	EAS	M	Blood	2 501 575	–	–	8697	6190	–	–	–	BN, IL, PB
Ebert *et al.* 2021 *Science* (124)	HG02492	SAS	M	LCL	3 565 097	372 637	347 792	13 993	8994	108	16.3	20.8	PB, S-seq
Chaisson *et al.* 2019 *Nat. Commun.* (141)	HG00733	AMR	F	LCL	–	343 950^h^	304 170^h^	16 566	10 607	128^i^	31.6	17.9^i^	10X, BN, Hi-C, IL, ON, PB, S-seq
Ebert *et al.* 2021 *Science* (124)	HG00731	AMR	M	LCL	3 693 860	379 989	379 972	14 009	8867	107	15.6	20.0	PB, S-seq

a We selected studies spanning the start of personal genome sequencing in 2007 until 2021, including those from diverse populations analyzed using different technologies. The size definitions used to categorize indels (insertions and deletions) and CNVs (insertions/duplications and deletions) varied between studies, leading to significant differences in numbers presented. The Levy *et al.* study (HuRef/Venter genome) provides a composite analysis, demonstrating that relative to the reference genome, ~ 1.3% of nucleotides were affected by indels and CNVs compared with 0.1% by SNVs. More recent studies further support the idea that non-SNV variation affects several times more nucleotides than SNVs (58,124,141). Where reported, balanced SVs (inversions in most studies) encompass between 9.3 and 22.2 Mb (average 18 Mb). Data from these studies are typically also accessible in public repositories (159,173–175).

b The total number of base pairs affected by non-SNV sequence changes. Unbalanced changes include insertions and deletions of all sizes, whereas balanced changes include inversions.

c Abbreviations: AFR, African; AMR, Admixed American; EAS, East Asian; EUR, European; SAS, South Asian.

d Additions of genetic material are typically described as insertions when detected by comparisons between assembled genomes and as duplications when detected using chromosomal microarray analysis.

e The technologies used for sequencing, assembly and variant detection. Abbreviations: 10x, 10x Genomics linked reads (60–62); 454, 454 Life Sciences pyrosequencing (32,33); BN, Bionano Genomics optical mapping (65); CMA, chromosomal microarray analysis (20–26); IL, Illumina (Solexa) sequencing (34); ON, Oxford Nanopore Technologies sequencing (137–140); PB, Pacific Biosciences sequencing (59); SOLiD, sequencing by oligonucleotide ligation and detection (35); SS, Sanger sequencing (49); S-seq, strand-seq (135,136).

f Values represent homozygous indels; 292 102 heterozygous indels (not stratified by insertions and deletions in the paper) were also detected.

g Insertions and deletions detected using assembly comparison are listed under indels, whereas those detected using other methods are listed under CNVs/SVs.

h Detected by Illumina sequencing.

i Reflects simple inversions as tabulated in Supplementary Table 9 of Chaisson *et al.* (141)

j Excludes indels.

k Composite of three different Japanese males.

LCL, lymphoblastoid cell line.

Additional early studies used direct (clones not required) massively parallel sequencing technologies to generate personal genome sequences for two other pioneers of genome research—James Watson and James Lupski, both of European ancestry (53,54). These million-dollar projects utilized 454 pyrosequencing (32,33) and massively parallel sequencing by ligation (35), yielding 3 322 093 and 3 420 306 SNVs, respectively, with only a few SVs being reported. Concurrently, using what would become a mainstay technology in genomics (Solexa, eventually becoming Illumina sequencing), Bentley *et al.* (34) analyzed the genome of a male Yoruban individual using massively parallel sequencing-by-synthesis. Their data revealed nearly 1 million more SNVs compared with the previously-mentioned genomes of individuals of European ancestry (50,53,54), as well as >400 000 indels and 5000 SVs, many of which were previously unknown. A separate analysis of African hunter-gatherers, the oldest lineages of modern humans, revealed a similar number of SNVs (~4 million) as reported by Bentley, with the trend being that more genetic variation tends to be found in ‘older’ populations [(55); Table 1].

Published in 2009, the sequencing of the first Korean genome (AK1) used an integrated approach with Illumina shotgun sequencing, bacterial artificial chromosome sequencing and CMA, reporting 3 453 653 SNVs, 170 202 indels and 1237 SVs (56). Interestingly, only 37% of the non-synonymous SNVs in AK1 were also found in both the previously-sequenced African (34) and Chinese (57) genomes. A *de novo* assembly of the AK1 genome with haplotype phasing was subsequently generated (58) using Pacific Biosciences (PacBio) long-read sequencing (59), Illumina short reads (34) and 10x Genomics linked-read technology (60–62). A similar number of SNVs were detected (3 472 576 versus 3 453 653) along with more refined SV data afforded by the long-read technology, including many sequences not found in the human reference genome. Other notable projects sequencing the genomes of individuals of Asian descent include a high-coverage phased Chinese genome [HX1; (63)] and a haploid Japanese genome reference assembled through the consensus among three donors (64) using high-coverage PacBio long reads (59) and Bionano Genomics optical mapping (65). Approximately 2.5 million new SNVs and over 14 000 SVs were reported in the composite Japanese genome, many of which were found to be common in the Japanese population (Table 1). The Japanese study also demonstrated that population-specific reference genomes may facilitate the identification of disease-associated variants compared with using the standard reference. Given that analysis pipelines often ignore sequence reads that do not map to the GRCh38 reference sequence, the construction of this and other population-specific reference genomes (66–69) will surely prove to be important in accurately capturing the full spectrum of DNA sequence (including complex and repetitive elements), as well as genetic variation, in diverse human populations. Additional strategies for improving the reference genome include adjusting all alleles to the major allele form (70).

## *De novo* Mutation Across a Generation

Cataloguing the nature and extent of inherited genetic variation in human populations is important from an evolutionary perspective (71–74), and determining the presence of new variants (*de novo* mutations or DNMs) is critical in medical genomics (75–77). Early estimates of mutation rates were made using cross-species comparisons (78), small numbers of human genetic loci (79) or—in a seminal paper in *Human Molecular Genetics*—specific tandem repeat loci (14). However, the direct measurement of genome-wide mutation rates requires WGS of biological parent–child trios, which has only become feasible at scale, with increasing completeness and accuracy, over the last 10 years. Therefore, the first such studies included small numbers of trios (80,81), with more recent studies involving orders of magnitude more families, often as part of disease studies (Table 2). For reasons of cost (a 30x coverage genome today at ~$1000) and accuracy (at least for SNVs), the sequencing method of choice has been Illumina short-read technology, so accordingly, most of the DNM data presented are limited to SNVs. As discussed in Table 1, comprehensive and accurate detection of larger variants is challenging with short-read data alone, so until recently, much of the information for *de novo* CNVs has come from CMA (Table 2).

**Table 2:** Important genome-wide studies examining *de novo* variation across a generation^a^

Study	Families	Phenotype^b^	Technology^c^	DNM rate (events/generation)^d^	Paternal age effect^e^	Maternal age effect^e^
Sebat *et al.* 2007 *Science* (105)	264	ASD	CMA	0.01 CNVs^f^	–	–
Itsara *et al.* 2010 *Genome Res.* (107)	2197	ASD	CMA	Varies by size^g^	–	–
Roach *et al.* 2010 *Science* (81)	1	See note^h^	WGS	70 SNVs	–	–
Conrad *et al.* 2011 *Nat. Genet.* (80)	2	NA	WGS	42 SNVs	–	–
Michaelson *et al.* 2012 *Cell* (82)	10	ASD	WGS	58 SNVs	1.0 SNVs	–
Kong *et al.* 2012 *Nature* (83)	78	ASD, SCZ	WGS	63 SNVs	2.0 SNVs	–
Campbell *et al.* 2012 *Nat. Genet.* (84)	5	NA	WGS	35 SNVs^i^	–	–
Gilissen *et al.* 2014 *Nature* (85)	50	ID	WGS	82 SNVs, 0.16 CNVs	–	–
Francioli *et al.* 2014, 2015 *Nat. Genet.* (86,87)	250	NA	WGS	43 SNVs	1.1 SNVs	–
Wong *et al.* 2016 *Nat. Commun.* (88)	693	PTB	WGS	39 SNVs	0.64 SNVs	0.35 SNVs
Goldmann *et al.* 2016 *Nat. Genet.* (89)	816	PTB	WGS	45 SNVs	0.91 SNVs	0.24 SNVs
Yuen *et al.* 2016 *NPJ Genom. Med.* (90)	200	ASD	WGS	51 SNVs, 4 indels, 0.05 CNVs^j^	–	–
Yuen *et al.* 2017 *Nat. Neurosci.* (91)	1239	ASD	WGS	74 SNVs, 13 indels	–	–
Jónsson *et al.* 2017 *Nature* (92)	1548	Various	WGS	65 SNVs, 5 indels	1.51 SNVs+indels	0.37 SNVs+indels
Maretty *et al.* 2017 *Nature* (93)	50	NA	WGS	64 SNVs, 6 indels	–	–
An *et al.* 2018 *Science* (95)	1902	ASD	WGS	62 SNVs, 6 indels	–	–
Kessler *et al.* 2020 *Proc. Natl. Acad. Sci.* (94)	1465	Various	WGS	64 SNVs	1.35 SNVs	0.42 SNVs
Collins *et al.* 2020 *Nature* (109)	970	Various	WGS	0.29 SVs^k^	–	–
Belyeu *et al.* 2021 *Am. J. Hum. Genet.* (110)	2396	ASD	WGS	0.16 SVs^l^	Not significant	Not significant
Mitra *et al.* 2021 *Nature* (103)	1637	ASD	WGS	53 tandem repeat indels^m^	Significant^n^	–

a We selected studies that tested for genome-wide *de novo* mutation events from population control or disease datasets. Each study has strengths and weaknesses in design, data capture and experimental validation. Four comprehensive studies (90–93) report an average of 64 SNV, 7 indel and 0.05 CNV events per generation.

b The phenotype or disease of participants in the study. ‘NA’ means that only healthy controls were used or that no disease phenotype was indicated. ASD, autism spectrum disorder; ID, intellectual disability; PTB, preterm birth; SCZ, schizophrenia.

c The technology used for variant detection. CMA, chromosomal microarray analysis; WGS, whole-genome sequencing.

d DNM rates are reported in terms of events per generation because this measure is generalizable across variant types (i.e. also including indels and SVs). As mentioned in the text, after adjusting for the proportion of the genome assessed, estimates of per-nucleotide mutation rates for *de novo* SNVs are consistently reported as ~1.2 × 10^−8^ per generation.

e The estimated number of additional *de novo* variants per year of parental age.

f CNVs > 99 kb in unaffected individuals only.

g CNVs > 30 kb: 0.012; CNVs > 500 kb: 0.0065.

h The two siblings in this study each had two recessive disorders.

i This study also estimated mutation rates based on heterozygous positions within autozygous segments, giving a per-nucleotide mutation rate of 1.2 × 10^−8^ per generation.

j CNVs > 10 kb.

k Includes 0.15 deletions, 0.1 insertions, 0.04 duplications and 0.001 inversions.

l Value is for healthy individuals; DNM rate was significantly higher in ASD-affected individuals (0.21 SVs/generation).

m Value is for healthy individuals; DNM rate was slightly but significantly higher in ASD-affected individuals (55 tandem repeat indels/generation).

n Paternal age effect was statistically significant, but no slope given.

Considering SNVs alone, studies have revealed 35–82 DNMs per generation within the mappable genome [(80–94); Table 2]. Although reasonably consistent, these estimates are not perfectly comparable across studies due to differences in the proportion of the genome assessed. After adjustment, studies consistently report a mutation rate of ~ 1.2 × 10^−8^ per nucleotide per generation (83,84,88,92–94). Interestingly, mutation rate estimates from trios are highly concordant with earlier estimates (78,79). By comparing DNMs in monozygotic twins, it has been estimated that ~97% are germline in origin, whereas 3% are somatic (87). Although some studies in Table 2 include individuals ascertained for specific diseases, little difference has been observed in the total number of constitutional *de novo* SNVs compared with healthy individuals (95).

Many DNM studies have examined the parental age effect—the number of additional DNMs per year of parental age. This effect is greater in fathers, with estimates ranging from 0.64 to 2.0 additional DNMs per additional year of age versus 0.24–0.42 for mothers (Table 2). As a result, fathers contribute more DNMs per generation than mothers; paternal/maternal ratios of 3–5 have been reported (83,84,88,92), an observation increasingly made in studies of autism (90,91,96,97). Although DNMs in general are more likely to be of paternal origin, some genomic regions exhibit a significant bias toward maternally-derived DNMs (89).

Although most DNM studies have examined homogeneous population groups [e.g. Dutch, Icelandic or Danish citizens; (87,92,93)] or have not investigated the effect of ancestry, one study found that mutation rates were generally consistent across populations, but were ~7% lower in Amish individuals (94). The same study found that the contribution of additive genetic effects to mutation rate is non-existent (94); thus, variation in mutation rate not explained by parental age is likely due to some combination of non-additive genetic effects and environmental factors. In the case of the Amish, it seems plausible that the observed difference could be partially accounted for by some combination of consanguinity and lifestyle factors, such as reduced exposure to mutagens.

Interestingly, WGS studies have revealed no clear impact of extreme environmental exposure on DNM rates, including in children of parents exposed to dioxin (98) or to radiation from the atomic bombings of Hiroshima and Nagasaki (99) or the Chernobyl nuclear accident (100).

DNMs do not occur with equal probability throughout the genome; rather, their frequency is influenced by sequence context. Trio studies have shown that ~2/3 of DNMs are transitions and that these events occur 20x more frequently at CpG sites (83). DNMs from younger fathers are more likely to occur in late-replicating genomic regions, whereas no such effect has been observed in mothers or older fathers (87). Because early-replicating regions are more gene-rich (101), this bias may further increase the probability of a deleterious DNM originating from an older father. Representing ~2% of all DNMs, DNM clusters have been observed, typically within 20 kb windows, and appear to have distinct mutational signatures compared with non-clustered DNMs (87,89). The number of DNM clusters increases with parental age at an approximately equal rate for mothers and fathers; this suggests that they arise from a different mutational mechanism (compared with non-clustered DNMs) that is common between mothers and fathers (89), although some differences in paternally- versus maternally-derived clusters have been observed (92). Studies of autism have also observed clustered DNMs (82,90), which are mainly maternally-derived and are often found adjacent to *de novo* CNVs (90). A comprehensive review of mutational patterns, as well as the disease implications of *de novo* variants, is published (102).

Recent studies have estimated that 4–13 *de novo* indels occur per generation (90–93,95). Deletions were found to be more common than insertions, and even-sized indels were more common than odd-sized indels (93). Specialized algorithms for identifying *de novo* indels within tandem repeat loci have detected ~55 events per genome in healthy individuals (103), along with a paternal origin bias and age effect. The corresponding tandem repeat *de novo* rate, estimated at 5.6 × 10^−5^ per generation per locus, is far lower than much earlier estimates for tandem repeats based on a few loci and PCR-based tests (14), reflecting changes in accuracy afforded by better technology and genome-wide genotyping ability. However, that so many *de novo* indels were detected in tandem repeat regions over and above those detected in non-repetitive regions suggests that the total degree of *de novo* variation has been underestimated—not only for indels, but also for other classes of variation shown in Figure 1. As new technologies and algorithms improve our ability to interrogate repetitive and difficult-to-map regions of the genome, measured *de novo* rates for all types of variation will rise.

Compared with SNVs and indels, *de novo* rates for CNVs and SVs have been less well-characterized. CMA has revealed that CNV mutation rates differ depending on CNV size and that large *de novo* CNVs are substantially more frequent in individuals with autism compared with unaffected individuals (104–107), some of which are recurrent and clinically relevant (108). Another autism study estimated the rate of *de novo* CNVs > 10 kb at 0.05 per generation (90). Recently, Collins *et al.* (109) used WGS to estimate mutation rates for SVs > 50 bp, with each generation averaging 0.15 *de novo* deletions, 0.1 insertions, 0.04 duplications and 0.001 inversions. Yet another recent study found ~0.16 *de novo* SVs per healthy individual, along with a significantly higher rate (0.21) in individuals with autism (110). Interestingly, the latter study found that most *de novo* SVs originated from the father but did not find statistical evidence for a parental age effect on *de novo* SV rate, which is in contrast to the well-established parental age effect for *de novo* SNVs (82,83,87–89,92,94).

## Redefining Genomic Variation Using Short- and Long-Read WGS

As affordable WGS has become commonplace, the ability to comprehensively detect the many classes of genetic variation in large, diverse sets of individuals (111–117) has improved considerably, aided by the development of variant benchmarking resources (118–120). These studies have, in turn, enabled the study of disease (109,121,122), human migration and adaptation patterns (123) and evolution (124). As genetic variation becomes better defined across different ancestry groups (93,125–128), including in archaic genomes (Denisova, Neanderthal) (129,130), an increasing amount of genetic variation is being found among lineages. Personal genome sequencing of diverse populations with different technologies is also revealing novel DNA sequences (and therefore genetic variation) not currently present in the human reference genome and corresponding databases (55,58,67,131). In perhaps the most astounding example of the power of sequencing technology to map variants across a generation, an ‘F1’ offspring of a *Homo sapiens neanderthalensis* and *Homo sapiens denisova* was discerned (132). Most of the aforementioned studies concentrate on SNVs, since they are the easiest to discover from the current industry-standard short-read sequencing technology.

Recently, papers describing ‘end-to-end’ chromosome assemblies have been published, focusing on using long-read sequencing technologies to enable SV discovery and mapping [Table 1; (133,134)]. In a *tour de force* effort, PacBio long-read (59) and strand-specific (135,136) sequencing technologies were used to generate haplotype-resolved *de novo* assemblies of 32 diverse individuals at an estimated cost per genome of ~$20 000 (124). With this approach, 107 590 SVs were found, representing an average of 16 Mb of structural variation per individual, of which 68% were not discovered using standard short-read sequencing. In a parallel effort using a multi-platform approach [PacBio (59) and Oxford Nanopore (137–140) long-read sequencing, Illumina short-read sequencing (34), 10x Genomics linked reads (60–62) and Bionano Genomics optical mapping (65)], three trios of Han Chinese, Puerto Rican and Yoruban ancestry were sequenced, yielding SV sets 3–7x larger than most other standards (141). As shown in Table 1, the unbalanced SVs impacted 31.6, 39.3 and 39.8 Mb in admixed American, East Asian and African ancestries, respectively, all closer to what was found using the integrated approach in the HuRef/Venter project (50,51). The impact of balanced inversions is also shown in Table 1. Although giving near chromosome-level resolution, these long-read sequencing studies emphasize limitations in assembly and discrimination, particularly at gene-rich regions harboring complex structural variation. Given the current error rate of these technologies, accurately detecting SNVs still requires ‘filling in’ using short-read sequence data, highlighted by the fact that some trio studies do not overtly report DNM rates or SNV quality (124,141). In studies using cell line-derived DNA, the transforming viral integration process and culturing can cause modest but detectable changes in the genome (142,143), which may also be a confounder.

Many studies, including one describing the use of Oxford Nanopore long-read technology to study the Icelandic population (117,144–148), reaffirm the need to consider large-scale copy number and structural variation in disease study design. In our own recent research, developing novel computational and statistical methods to analyze existing short-read sequence data for expanded tandem repeats led to the discovery of specific loci associated with autism (149), an intriguing finding given that most known disorders associated with tandem repeat expansions are monogenic (150). The same study also discovered extensive polymorphism in repeat motif size and sequence, often correlated with cytogenetic ‘fragile site’ variation along chromosomes (149). Moreover, 158 991 ultra-rare SVs were recently found through the study of 17 795 population controls, with 2% of individuals carrying megabase-scale SVs (117). The same study found reciprocal translocations at a rate of 1 in 1000 individuals, a number similar to that found using classical cytogenetics (151,152).

There are two fundamental steps to identifying associations between genotypes and health: variant detection and variant interpretation. With the combination of long-read technology and other sequencing methods now enabling the ‘complete’ sequencing of chromosomes (133,134,153), making further improvements for variant detection essentially represents an engineering problem. Although significant challenges remain, including cost reductions in long-read sequencing, accurate phasing of diploid genomes and scaling the end-to-end assembly process to entire populations, it seems plausible that variant detection will eventually become a *fait accompli*. To the contrary, variant interpretation is still in its early days, perhaps even reminiscent of examining chromosome banding in the 1960s (154–158). Although our ability to interpret the impact of copy number changes and loss-of-function sequence-level variants is somewhat mature, understanding the effects of most other alterations, such as missense variants and variants impacting regulatory elements, remains largely unresolved. The rapidly increasing pace by which sequencing data are now generated, along with the move to examining populations at scale and the use of multi-omics technologies, ultimately promise to reduce the time from data generation to data interpretation (159–167).

## Conclusions

The current assembly of the human genome (GRCh38) comprises 3 099 706 404 bp. Comparing any other genome to it yields ~3–4 million SNVs and (with comprehensive multi-technology testing) ~10 times as many nucleotides impacted by unbalanced structural variations, most notably indels and CNVs (Table 1). Notwithstanding the many complexities in whole-genome analysis, it can be conservatively stated that ~ 1% variation exists between each of our DNA when compared with the reference, with those genomes arising from African and other ancestral populations exhibiting more genetic variation than those arising more recently in human history. A consistent message from the literature is that no single technology or method can detect all genetic variation, and knowledge of how the data (and databases housing it) were derived is essential to correctly interpreting it. The number of DNMs found in the mappable euchromatic DNA in a single individual is modest (fewer than 100), but this value may increase as more complex sequences are considered in tallies of genetic variation—noting, however, that nomenclature and reporting of SVs, in particular in repetitive regions, is challenging (159,168–170). Newer WGS technologies (e.g. long-read sequencing) that facilitate the discovery and genotyping of complex variants will have a growing impact in disease studies and population sequencing as their costs begin to compete with the more prevalent short-read technologies. When analyzing larger sample sizes for their genomic architecture, cost considerations mean that short-read sequencing studies will prevail, likely for a while, even when considering structural variation. Drawing from the fundamental genomic data presented in Tables 1 and 2, we calculate that from 4 billion births (171) and ~71 *de novo* SNVs/indels/CNVs per individual, >284 billion DNMs have arisen over the past 30 years of human history. Such a wellspring of genetic variation, once characterized, will power the next generation of studies in human molecular genetics.
